# Supplementary material for: The structure and dynamics of secretory component and its interactions with polymeric immunoglobulins
Source: eLife. 2016 Mar 4;5:e10640. doi: 10.7554/eLife.10640 (PMC4786434; doi:10.7554/eLife.10640)
Supplement: Supplementary file 1. — Values in parenthesis refer to the highest-resolution shell. aR-meas, redundancy-independent merging R-factor (Diederichs and Karplus, 1997). bCC1/2, correlation of one half of the reflections to the other half. cCC*, CC1/2 modification showing the correlation of the observed data to unknown true intensities (Karplus and Diederichs, 2012). dRcryst = (Σ Fobs-Fcalc)/(Σ Fobs) and Rfree = Rcryst calculated for 5–10% of reflections from each structure that were excluded from refinement. eRMS = Root mean square deviation from ideal value. fDetermined by Molprobity (Chen et al., 2010). DOI: http://dx.doi.org/10.7554/eLife.10640.016 [file elife-10640-supp1.docx]

**Supplementary File 1: Crystallographic Data Collection and Refinement Statistics.**

|  | **hSC** | **tSC (NaI)** | **tSC (native)** |
| --- | --- | --- | --- |
| **DATA COLLECTION** |  |  |  |
| Wavelength (Å) | 0.9537 | 1.7711 | 0.9537 |
| Resolution range (Å) | 36.98 - 2.60 (2.69 - 2.60) | 38.75 - 2.08 (2.19-2.08) | 38.04-1.75 (1.81-1.75) |
| Space group | P 2_1_ | P 4_3_ 2_1_ 2 | P 4_3_ 2_1_ 2 |
| Unit cell Dimensions (Å) | 61.26 242.43 63.05 β = 114.89˚ | 54.80 54.80 187.13 | 54.94 54.94 187.26 |
| Total reflections | 145929 (14232) | 272149 (33267) | 232192 (23342) |
| Unique reflections | 49197 (4025) | 32493 (4946) | 30018 (2920) |
| Multiplicity | 3.0 (2.9) | 2.0 (1.8) | 7.7 (8.0) |
| Completeness (%) | 91.0 (95.0) | 98.9 (93.5) | 99.81 (98.95) |
| Mean I/sigma (I) | 14.34 (1.91) | 11.45 (1.81) | 18.17 (1.90) |
| Wilson B-factor (Å^2^) | 51.52 | 30.1 | 35.95 |
| R-meas^a^ | 0.07026 (0.7481) | 0.136 | 0.05805 (1.122) |
| CC1/2^b^ | 0.998 (0.755) | 0.997 (0.571) | 0.999 (0.697) |
| CC*^c^ | 1 (0.927) | N/D | 1 (0.906) |
| **REFINEMENT** |  |  |  |
| R-cryst/R-free^d^ | 0.201/0.254 |  | 0.1851/0.2148 |
| Number of non-hydrogen atoms | 8545 |  | 1745 |
| macromolecules | 8320 |  | 1614 |
| ligands | 152 |  | N/A |
| Protein residues | 1075 |  | 206 |
| RMS^e^ (bonds) (Å) | 0.01 |  | 0.014 |
| RMS^e^ (angles) ˚ | 1.92 |  | 1.22 |
| Ramachandran favored (%) | 95 |  | 98 |
| Ramachandran outliers (%) | 0.0 |  | 0 |
| Clashscore^f^ | 3.24 |  | 2.21 |
| Average B-factor (Å^2^) | 63.12 |  | 41.54 |
| macromolecules | 62.69 |  | 41.22 |
| ligands | 92.01 |  | N/A |
| solvent | 48.94 |  | 45.43 |

**Supplementary Table 1. Crystallographic Data Collection and Refinement Statistics.** Values in parenthesis refer to the highest-resolution shell. **^a^R-meas**, redundancy-independent merging R-factor ([Diederichs and Karplus, 1997](#_ENREF_18))**. ^b^CC1/2,** correlation of one half of the reflections to the other half. **^c^CC*,** CC1/2 modification showing the correlation of the observed data to unknown true intensities ([Karplus and Diederichs, 2012](#_ENREF_35)). ^d^Rcryst = (Σ|Fobs-Fcalc|)/(Σ|Fobs)| and Rfree = Rcryst calculated for 5-10% of reflections from each structure that were excluded from refinement. ^e^RMS = Root mean square deviation from ideal value. ^f^Determined by Molprobity ([Chen et al., 2010](#_ENREF_12)).
