# Supplementary material for: The structure and dynamics of secretory component and its interactions with polymeric immunoglobulins
Source: eLife. 2016 Mar 4;5:e10640. doi: 10.7554/eLife.10640 (PMC4786434; doi:10.7554/eLife.10640)
Supplement: Supplementary file 2. — Table listing the name, residue numbers, mutation(s) (endogenous sequence -> mutated sequence) and mature protein sequence for all protein expression constructs used in this study. Residue numbering is based on the mature hSC and tSC sequences and abbreviations used are: SP (Signal peptide), 6HIS (hexahistidine affinity tag), Strep II (Strep II affinity tag), C->A (C468A; C502A mutation). DOI: http://dx.doi.org/10.7554/eLife.10640.017 [file elife-10640-supp2.docx]

**Supplementary File 2: Expression Constructs Used in This Study**

| **Construct ID** | **Residues** | **Mutations** | **Mature Protein Sequence** |
| --- | --- | --- | --- |
| hSC D1-D5 | SP-1-547-linker-StrepII |  | KSPIFGPEEVNSVEGNSVSITCYYPPTSVNRHTRKYWCRQGARGGCITLISSEGYVSSKY  AGRANLTNFPENGTFVVNIAQLSQDDSGRYKCGLGINSRGLSFDVSLEVSQGPGLLNDTK  VYTVDLGRTVTINCPFKTENAQKRKSLYKQIGLYPVLVIDSSGYVNPNYTGRIRLDIQGT  GQLLFSVVINQLRLSDAGQYLCQAGDDSNSNKKNADLQVLKPEPELVYEDLRGSVTFHCA  LGPEVANVAKFLCRQSSGENCDVVVNTLGKRAPAFEGRILLNPQDKDGSFSVVITGLRKE  DAGRYLCGAHSDGQLQEGSPIQAWQLFVNEESTIPRSPTVVKGVAGSSVAVLCPYNRKES  KSIKYWCLWEGAQNGRCPLLVDSEGWVKAQYEGRLSLLEEPGNGTFTVILNQLTSRDAGF  YWCLTNGDTLWRTTVEIKIIEGEPNLKVPGNVTAVLGETLKVPCHFPCKFSSYEKYWCKW  NNTGCQALPSQDEGPSKAFVNCDENSRLVSLTLNLVTRADEGWYWCGVKQGHFYGETAAV  YVAVEERGSWSHPQFEK |
| hSC D1-D5 C->A | SP-1-547-linker-StrepII | C468A  C502A | KSPIFGPEEVNSVEGNSVSITCYYPPTSVNRHTRKYWCRQGARGGCITLISSEGYVSSKY  AGRANLTNFPENGTFVVNIAQLSQDDSGRYKCGLGINSRGLSFDVSLEVSQGPGLLNDTK  VYTVDLGRTVTINCPFKTENAQKRKSLYKQIGLYPVLVIDSSGYVNPNYTGRIRLDIQGT  GQLLFSVVINQLRLSDAGQYLCQAGDDSNSNKKNADLQVLKPEPELVYEDLRGSVTFHCA  LGPEVANVAKFLCRQSSGENCDVVVNTLGKRAPAFEGRILLNPQDKDGSFSVVITGLRKE  DAGRYLCGAHSDGQLQEGSPIQAWQLFVNEESTIPRSPTVVKGVAGSSVAVLCPYNRKES  KSIKYWCLWEGAQNGRCPLLVDSEGWVKAQYEGRLSLLEEPGNGTFTVILNQLTSRDAGF  YWCLTNGDTLWRTTVEIKIIEGEPNLKVPGNVTAVLGETLKVPCHFPAKFSSYEKYWCKW  NNTGCQALPSQDEGPSKAFVNADENSRLVSLTLNLVTRADEGWYWCGVKQGHFYGETAAV  YVAVEERGSWSHPQFEK |
| hSC D1-D5 C->A | SP-1-547-linker-6HIS | C468A  C502A | KSPIFGPEEVNSVEGNSVSITCYYPPTSVNRHTRKYWCRQGARGGCITLISSEGYVSSKY  AGRANLTNFPENGTFVVNIAQLSQDDSGRYKCGLGINSRGLSFDVSLEVSQGPGLLNDTK  VYTVDLGRTVTINCPFKTENAQKRKSLYKQIGLYPVLVIDSSGYVNPNYTGRIRLDIQGT  GQLLFSVVINQLRLSDAGQYLCQAGDDSNSNKKNADLQVLKPEPELVYEDLRGSVTFHCA  LGPEVANVAKFLCRQSSGENCDVVVNTLGKRAPAFEGRILLNPQDKDGSFSVVITGLRKE  DAGRYLCGAHSDGQLQEGSPIQAWQLFVNEESTIPRSPTVVKGVAGSSVAVLCPYNRKES  KSIKYWCLWEGAQNGRCPLLVDSEGWVKAQYEGRLSLLEEPGNGTFTVILNQLTSRDAGF  YWCLTNGDTLWRTTVEIKIIEGEPNLKVPGNVTAVLGETLKVPCHFPAKFSSYEKYWCKW  NNTGCQALPSQDEGPSKAFVNADENSRLVSLTLNLVTRADEGWYWCGVKQGHFYGETAAV  YVAVEERGSHHHHHH |
| hSC D1 | SP-1-109-linker-StrepII |  | KSPIFGPEEVNSVEGNSVSITCYYPPTSVNRHTRKYWCRQGARGGCITLISSEGYVSSKY  AGRANLTNFPENGTFVVNIAQLSQDDSGRYKCGLGINSRGLSFDVSLEVGSWSHPQFEK |
| hSC D1-D3 | SP-1-329-linker-StrepII |  | KSPIFGPEEVNSVEGNSVSITCYYPPTSVNRHTRKYWCRQGARGGCITLISSEGYVSSKY  AGRANLTNFPENGTFVVNIAQLSQDDSGRYKCGLGINSRGLSFDVSLEVSQGPGLLNDTK  VYTVDLGRTVTINCPFKTENAQKRKSLYKQIGLYPVLVIDSSGYVNPNYTGRIRLDIQGT  GQLLFSVVINQLRLSDAGQYLCQAGDDSNSNKKNADLQVLKPEPELVYEDLRGSVTFHCA  LGPEVANVAKFLCRQSSGENCDVVVNTLGKRAPAFEGRILLNPQDKDGSFSVVITGLRKE  DAGRYLCGAHSDGQLQEGSPIQAWQLFVNGSWSHPQFEK |
| hSC D2-D3 | SP-110-329-linker-StrepII |  | SQGPGLLNDTKVYTVDLGRTVTINCPFKTENAQKRKSLYKQIGLYPVLVIDSSGYVNPNY  TGRIRLDIQGTGQLLFSVVINQLRLSDAGQYLCQAGDDSNSNKKNADLQVLKPEPELVYE  DLRGSVTFHCALGPEVANVAKFLCRQSSGENCDVVVNTLGKRAPAFEGRILLNPQDKDGS  FSVVITGLRKEDAGRYLCGAHSDGQLQEGSPIQAWQLFVNGSWSHPQFEK |
| hSC D1-D4-D5 | SP-1-111,  331-547-linker-StepII |  | KSPIFGPEEVNSVEGNSVSITCYYPPTSVNRHTRKYWCRQGARGGCITLISSEGYVSSKY  AGRANLTNFPENGTFVVNIAQLSQDDSGRYKCGLGINSRGLSFDVSLEVSQESTIPRSPT  VVKGVAGSSVAVLCPYNRKESKSIKYWCLWEGAQNGRCPLLVDSEGWVKAQYEGRLSLLE  EPGNGTFTVILNQLTSRDAGFYWCLTNGDTLWRTTVEIKIIEGEPNLKVPGNVTAVLGET  LKVPCHFPCKFSSYEKYWCKWNNTGCQALPSQDEGPSKAFVNCDENSRLVSLTLNLVTRA  DEGWYWCGVKQGHFYGETAAVYVAVEERGSWSHPQFEK |
| hSC  D1-D4-D5 C->A | SP-1-111,  331-547-linker-StepII | C468A  C502A | KSPIFGPEEVNSVEGNSVSITCYYPPTSVNRHTRKYWCRQGARGGCITLISSEGYVSSKY  AGRANLTNFPENGTFVVNIAQLSQDDSGRYKCGLGINSRGLSFDVSLEVSQESTIPRSPT  VVKGVAGSSVAVLCPYNRKESKSIKYWCLWEGAQNGRCPLLVDSEGWVKAQYEGRLSLLE  EPGNGTFTVILNQLTSRDAGFYWCLTNGDTLWRTTVEIKIIEGEPNLKVPGNVTAVLGET  LKVPCHFPAKFSSYEKYWCKWNNTGCQALPSQDEGPSKAFVNADENSRLVSLTLNLVTRA  DEGWYWCGVKQGHFYGETAAVYVAVEERGSWSHPQFEK |
| hSC  D1-D3-D4-D5  C->A | SP+ 1-117  223-547-linker-HIS | C468A  C502A | KSPIFGPEEVNSVEGNSVSITCYYPPTSVNRHTRKYWCRQGARGGCITLISSEGYVSSKY  AGRANLCNFPENGTFVVNIAQLSQDDSGRYKCGLGINSRGLSFDVSLEVSQGPGLLNEPE  LVYEDLRGSVTFHCALGPEVANVAKFLCRQSSGENCDVVVNTLGKRAPAFEGRILLNPQD  KDGSFSVVITGLRKEDAGRYLCGAHSDGQLQEGSPIQAWQLFVNEESTIPRSPTVVKGVA  GSSVAVLCPYNRKESKSIKYWCLWEGAQNGRCPLLVDSEGWVKAQYEGRLSLLEEPGNGT  FTVILNQLTSRDAGFYWCLTNGDTLWRTTVEIKIIEGEPNLKVPGNVTAVLGETLKVPCH  FPAKFSSYEKYWCKWNNTGCQALPSCDEGPSKAFVNADENSRLVSLTLNLVTRADEGWYW  CGVKQGHFYGETAAVYVAVEERGSHHHHHH |
| hSC D2-D5 C->A | SP-110-547-linker-StrepII | C468A  C502A | SQGPGLLNDTKVYTVDLGRTVTINCPFKTENAQKRKSLYKQIGLYPVLVIDSSGYVNPNY  TGRIRLDIQGTGQLLFSVVINQLRLSDAGQYLCQAGDDSNSNKKNADLQVLKPEPELVYE  DLRGSVTFHCALGPEVANVAKFLCRQSSGENCDVVVNTLGKRAPAFEGRILLNPQDKDGS  FSVVITGLRKEDAGRYLCGAHSDGQLQEGSPIQAWQLFVNEESTIPRSPTVVKGVAGSSV  AVLCPYNRKESKSIKYWCLWEGAQNGRCPLLVDSEGWVKAQYEGRLSLLEEPGNGTFTVI  LNQLTSRDAGFYWCLTNGDTLWRTTVEIKIIEGEPNLKVPGNVTAVLGETLKVPCHFPCK  FSSYEKYWCKWNNTGCQALPSQDEGPSKAFVNCDENSRLVSLTLNLVTRADEGWYWCGVK  QGHFYGETAAVYVAVEERGSWSHPQFEK |
| hSC D4-D5 C->A | SP-332-547-linker-StrepII | C468A  C502A | STIPRSPTVVKGVAGSSVAVLCPYNRKESKSIKYWCLWEGAQNGRCPLLVDSEGWVKAQY  EGRLSLLEEPGNGTFTVILNQLTSRDAGFYWCLTNGDTLWRTTVEIKIIEGEPNLKVPGN  VTAVLGETLKVPCHFPAKFSSYEKYWCKWNNTGCQALPSQDEGPSKAFVNADENSRLVSL  TLNLVTRADEGWYWCGVKQGHFYGETAAVYVAVEERGSWSHPQFEK |
| D1-D5  CDR1 Chimera | SP-1-X-547-linker-StrepII | FPCKFSSY ->  YSQGYRQH  C502A | KSPIFGPEEVNSVEGNSVSITCYYPPTSVNRHTRKYWCRQGARGGCITLISSEGYVSSKY  AGRANLTNFPENGTFVVNIAQLSQDDSGRYKCGLGINSRGLSFDVSLEVSQGPGLLNDTK  VYTVDLGRTVTINCPFKTENAQKRKSLYKQIGLYPVLVIDSSGYVNPNYTGRIRLDIQGT  GQLLFSVVINQLRLSDAGQYLCQAGDDSNSNKKNADLQVLKPEPELVYEDLRGSVTFHCA  LGPEVANVAKFLCRQSSGENCDVVVNTLGKRAPAFEGRILLNPQDKDGSFSVVITGLRKE  DAGRYLCGAHSDGQLQEGSPIQAWQLFVNEESTIPRSPTVVKGVAGSSVAVLCPYNRKES  KSIKYWCLWEGAQNGRCPLLVDSEGWVKAQYEGRLSLLEEPGNGTFTVILNQLTSRDAGF  YWCLTNGDTLWRTTVEIKIIEGEPNLKVPGNVTAVLGETLKVPCHYSQGYRQHEKYWCKW  NNTGCQALPSQDEGPSKAFVNADENSRLVSLTLNLVTRADEGWYWCGVKQGHFYGETAAV  YVAVEERGSWSHPQFEK |
| D1-D5  DE Loop Chimera | SP-1-X-547-linker-StrepII | GPSKAFVNCDENSR->  DDLTKA  C468A | KSPIFGPEEVNSVEGNSVSITCYYPPTSVNRHTRKYWCRQGARGGCITLISSEGYVSSKY  AGRANLTNFPENGTFVVNIAQLSQDDSGRYKCGLGINSRGLSFDVSLEVSQGPGLLNDTK  VYTVDLGRTVTINCPFKTENAQKRKSLYKQIGLYPVLVIDSSGYVNPNYTGRIRLDIQGT  GQLLFSVVINQLRLSDAGQYLCQAGDDSNSNKKNADLQVLKPEPELVYEDLRGSVTFHCA  LGPEVANVAKFLCRQSSGENCDVVVNTLGKRAPAFEGRILLNPQDKDGSFSVVITGLRKE  DAGRYLCGAHSDGQLQEGSPIQAWQLFVNEESTIPRSPTVVKGVAGSSVAVLCPYNRKES  KSIKYWCLWEGAQNGRCPLLVDSEGWVKAQYEGRLSLLEEPGNGTFTVILNQLTSRDAGF  YWCLTNGDTLWRTTVEIKIIEGEPNLKVPGNVTAVLGETLKVPCHFPAKFSSYEKYWCKW  NNTGCQALPSQDEDDLTKALVSLTLNLVTRADEGWYWCGVKQGHFYGETAAVYVAVEERG  SWSHPQFEK |
| D1-D5  CDR1-DE Chimera | SP-1-X-X-547-linker-StrepII | FPCKFSSY ->  YSQGYRQH  GPSKAFVNCDENSR->  DDLTKA | KSPIFGPEEVNSVEGNSVSITCYYPPTSVNRHTRKYWCRQGARGGCITLISSEGYVSSKY  AGRANLTNFPENGTFVVNIAQLSQDDSGRYKCGLGINSRGLSFDVSLEVSQGPGLLNDTK  VYTVDLGRTVTINCPFKTENAQKRKSLYKQIGLYPVLVIDSSGYVNPNYTGRIRLDIQGT  GQLLFSVVINQLRLSDAGQYLCQAGDDSNSNKKNADLQVLKPEPELVYEDLRGSVTFHCA  LGPEVANVAKFLCRQSSGENCDVVVNTLGKRAPAFEGRILLNPQDKDGSFSVVITGLRKE  DAGRYLCGAHSDGQLQEGSPIQAWQLFVNEESTIPRSPTVVKGVAGSSVAVLCPYNRKES  KSIKYWCLWEGAQNGRCPLLVDSEGWVKAQYEGRLSLLEEPGNGTFTVILNQLTSRDAGF  YWCLTNGDTLWRTTVEIKIIEGEPNLKVPGNVTAVLGETLKVPCHYSQGYRQHEKYWCKW  NNTGCQALPSQDEDDLTKALVSLTLNLVTRADEGWYWCGVKQGHFYGETAAVYVAVEERG  SWSHPQFEK |
| hSC  D1-D5 C->A  T67C Q491C | SP+1-547-linker-6HIS | C468A  C502A  T67C  Q491C | KSPIFGPEEVNSVEGNSVSITCYYPPTSVNRHTRKYWCRQGARGGCITLISSEGYVSSKY  AGRANLCNFPENGTFVVNIAQLSQDDSGRYKCGLGINSRGLSFDVSLEVSQGPGLLNDTK  VYTVDLGRTVTINCPFKTENAQKRKSLYKQIGLYPVLVIDSSGYVNPNYTGRIRLDIQGT  GQLLFSVVINQLRLSDAGQYLCQAGDDSNSNKKNADLQVLKPEPELVYEDLRGSVTFHCA  LGPEVANVAKFLCRQSSGENCDVVVNTLGKRAPAFEGRILLNPQDKDGSFSVVITGLRKE  DAGRYLCGAHSDGQLQEGSPIQAWQLFVNEESTIPRSPTVVKGVAGSSVAVLCPYNRKES  KSIKYWCLWEGAQNGRCPLLVDSEGWVKAQYEGRLSLLEEPGNGTFTVILNQLTSRDAGF  YWCLTNGDTLWRTTVEIKIIEGEPNLKVPGNVTAVLGETLKVPCHFPAKFSSYEKYWCKW  NNTGCQALPSCDEGPSKAFVNADENSRLVSLTLNLVTRADEGWYWCGVKQGHFYGETAAV  YVAVEERGSHHHHHH |
| hSC  D1-D5 C->A  T67C V455C | SP+1-547-linker-6HIS | C468A  C502A  T67C  V455C | KSPIFGPEEVNSVEGNSVSITCYYPPTSVNRHTRKYWCRQGARGGCITLISSEGYVSSKY  AGRANLCNFPENGTFVVNIAQLSQDDSGRYKCGLGINSRGLSFDVSLEVSQGPGLLNDTK  VYTVDLGRTVTINCPFKTENAQKRKSLYKQIGLYPVLVIDSSGYVNPNYTGRIRLDIQGT  GQLLFSVVINQLRLSDAGQYLCQAGDDSNSNKKNADLQVLKPEPELVYEDLRGSVTFHCA  LGPEVANVAKFLCRQSSGENCDVVVNTLGKRAPAFEGRILLNPQDKDGSFSVVITGLRKE  DAGRYLCGAHSDGQLQEGSPIQAWQLFVNEESTIPRSPTVVKGVAGSSVAVLCPYNRKES  KSIKYWCLWEGAQNGRCPLLVDSEGWVKAQYEGRLSLLEEPGNGTFTVILNQLTSRDAGF  YWCLTNGDTLWRTTVEIKIIEGEPNLKVPGNVTACLGETLKVPCHFPAKFSSYEKYWCKW  NNTGCQALPSQDEGPSKAFVNADENSRLVSLTLNLVTRADEGWYWCGVKQGHFYGETAAV  YVAVEERGSHHHHHH |
| hSC  D1-D5 C->A  V455C Q491C | SP+1-547-linker-6HIS | C468A  C502A  V455C  Q491C | KSPIFGPEEVNSVEGNSVSITCYYPPTSVNRHTRKYWCRQGARGGCITLISSEGYVSSKY  AGRANLTNFPENGTFVVNIAQLSQDDSGRYKCGLGINSRGLSFDVSLEVSQGPGLLNDTK  VYTVDLGRTVTINCPFKTENAQKRKSLYKQIGLYPVLVIDSSGYVNPNYTGRIRLDIQGT  GQLLFSVVINQLRLSDAGQYLCQAGDDSNSNKKNADLQVLKPEPELVYEDLRGSVTFHCA  LGPEVANVAKFLCRQSSGENCDVVVNTLGKRAPAFEGRILLNPQDKDGSFSVVITGLRKE  DAGRYLCGAHSDGQLQEGSPIQAWQLFVNEESTIPRSPTVVKGVAGSSVAVLCPYNRKES  KSIKYWCLWEGAQNGRCPLLVDSEGWVKAQYEGRLSLLEEPGNGTFTVILNQLTSRDAGF  YWCLTNGDTLWRTTVEIKIIEGEPNLKVPGNVTACLGETLKVPCHFPAKFSSYEKYWCKW  NNTGCQALPSCDEGPSKAFVNADENSRLVSLTLNLVTRADEGWYWCGVKQGHFYGETAAV  YVAVEERGSHHHHHH |
| tSC  D1-D2 | SP+1-213-6HIS |  | RVTTVGDLAVLEGRSVMIPCHYGPQYASYVKYWCRGSVKDLCTSLVRSDAPRGPAAAGED  KVVMFDDPVQQVFTVTMTELQKEDSGWYWCGVEVGGVWSADVTASLHINVIQGLSVVNSM  VSGEEGTSVTVQCLYSQGYRQHEKRWCRSGDWSSCLVTDGEGRYEDQAVEIRDDLTKAFT  VTLKGLARRDTGWYWCAAGQQQVAVYILVTPPSHHHHHH |

**Supplementary Table 2. Expression Constructs Used in This Study.** Table listing the name, residue numbers, mutation(s) (endogenous sequence -> mutated sequence) and mature protein sequence for all protein expression constructs used in this study. Residue numbering is based on the mature hSC D1-D5 and tSC sequences and abbreviations used are: SP (Signal peptide), 6HIS (hexahistidine affinity tag), Strep II (Strep II affinity tag), C->A (C468A; C502A mutation).
